# PM 2.5 juvenile exposure–induced spermatogenesis dysfunction by triggering testes ferroptosis and antioxidative vitamins intervention in adult male rats

**DOI:** 10.1007/s11356-023-30150-2

**Published:** 2023-10-06

**Authors:** Xiang Liu, Yaya Ai, Mingchen Xiao, Cao Wang, Zhen Shu, Jia Yin, Yu Chu, Qing Xiao, Bin Liu

**Affiliations:** 1 Department of Pediatric Surgery, Affiliated Hospital of Zunyi Medical University, Zunyi, Guizhou Province China; 2 Guizhou Children’s Hospital, Zunyi, Guizhou Province China; 3 Suining Central Hospital, Suining, Sichuan Province China; 4 Department of Pediatric Surgery, Longgang District Maternity & Child Healthcare Hospital of Shenzhen City, Shenzhen, Guangdong Province 518100 China; 5 Department of Pediatric Surgery, Longgang Maternity and Child Institute of Shantou University Medical College, Shenzhen, Guangdong Province 518100 China

**Keywords:** Automobile exhaust, PM2.5, Ferroptosis, Reproductive dysfunction, Redox balance, Antioxidative vitamins intervention

## Abstract

**Abstract:**

PM2.5 derived from automobile exhaust can cause reproductive impairment in adult males, but the toxic effects of PM2.5 exposure on reproductive function in juvenile male rats and its relationship with ferroptosis have not been reported. In this paper, 30-day-old juvenile male Sprague-Dawley (SD) rats were divided into four groups (blank control, vitamin control, PM2.5, and PM2.5+Vitamin). The blank control group was fed normally, and the vitamin control group was given intragastric administration of vitamins in addition to normal feeding. PM2.5 was administered via tracheal intubation. When the rats were treated for 4 weeks until reaching the period of sexual maturity. A mating test was performed first, and then their testicular and epididymal tissues were studied. Compared with control rats, juvenile male rats exposed to PM2.5 showed a decreased sperm count and fertility rate, redox imbalance, damaged mitochondria, a metabolic disorder of intracellular iron ions, and a significant rise in ferroptosis during the period of sexual maturity. After antioxidative vitamins intervention, the redox imbalance, metabolic disorder of intracellular iron ions, and ferroptosis were all alleviated, leading to the following conclusions: after being exposed to PM2.5 from automobile exhaust, male juvenile rats during the period of sexual maturity have significantly decreased reproductive function. The reproductive toxicity of PM2.5 is closely related to oxidative stress and ferroptosis. In addition, ferroptosis decreases and reproductive function is recovered to some degree after antioxidative vitamins intervention.

**Graphical abstract:**

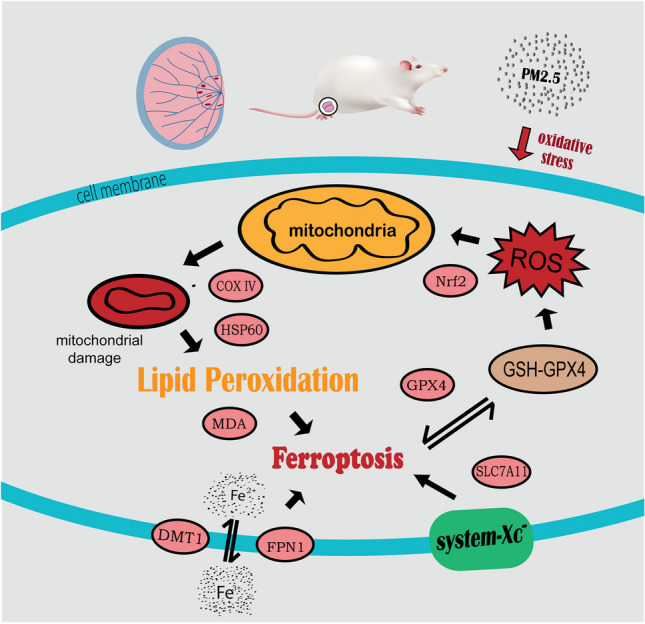

## Introduction

Air pollution is ubiquitous and seriously jeopardizes human health (Lelieveld et al. 2015). According to the Health Effects Research Institute (HERI), air pollution is the fifth leading risk factor for unnatural death in humans (Sorensen et al. 2022), and air pollution can cause and exacerbate a variety of health problems including cardiovascular and respiratory diseases, allergic diseases, vector-borne illnesses, pregnancy complications, and mental health disorders (Landrigan and Grandjean 2021; Shah et al. 2013; Solomon et al. 2022), and children are also deeply affected by environmental pollution’s adverse effects of environmental pollution (Nadeau et al. 2022; Perera and Nadeau 2022; Rajagopalan and Narula 2022). It is estimated that 8.7 million people die each year from diseases caused by particulate matter released into the air from the combustion of fossil fuels (Vohra et al. 2021).

Automobile exhaust seriously endangers reproductive health, and exposure to toxic pollutants like PM2.5 increases infertility risk (Zhang et al. 2018; Zhou et al. 2020). PM2.5 from automobile exhaust induces blood-testis barrier disruption in adult male testicular Sertoli cells through the ROS-MAPK-Nrf2 pathway, which in turn leads to impaired spermatogenesis (Liu et al. 2020; Liu et al. 2019). Immature testes are more susceptible to these effects due to incomplete development and lower resistance to external invasion, and adult male reproductive disorders may be related to immature testicular developmental damage (Nadeau et al. 2022; Perera and Nadeau 2022; Skakkebaek et al. 2016). Thus, elucidating the specific mechanism underlying PM2.5 damage to immature testicular development has significant value for the early prevention and treatment of male infertility and the improvement of fertility.

Oxidative stress has been proven to act as the “initiating agent” promoting the induction of reproductive impairment by PM2.5 (Liu et al. 2020; Liu et al. 2019). Moreover, mitochondria serve as the center of cell energy metabolism, the core organelle controlling signals for cellular intrinsic apoptosis, and an important organelle for generating and regulating reactive oxygen species (ROS), and they are vulnerable to environmental toxins (Calvo et al. 2016; Qureshi et al. 2017). Ferroptosis is triggered when mitochondrial dysfunction occurs and the excessive accumulation of lipid ROS exceeds the redox content maintained by the organism (Gao et al. 2019).

Ferroptosis is an iron ion–dependent form of cell death triggered by the toxic overaccumulation of lipid peroxides on cell membranes, and its pathogenesis and resulting morphology differ from those of other forms of cell death such as apoptosis and autophagy (Dixon et al. 2012). Several authors have confirmed that PM2.5 can cause damage to various systems, with ferroptosis being observed in the circulatory, respiratory, and digestive systems (Gu et al. 2023; Guohua et al. 2021; Hu et al. 2023; Park et al. 2023; Wang and Tang 2019). The mechanisms associated with ferroptosis are widely recognized as iron overload, lipid peroxidation, and the dysregulation of System Xc^−^ activity; reproductive system damage is also strongly associated with ferroptosis (Li et al. 2018).

Iron overload was found to induce granulosa cell ferroptosis and the release of granulosa cell exosomes containing abnormal miRNAs that impair oocyte development and maturation (Ni et al. 2022). Ferroptosis of testicular spermatogenic cells in adult mice is due to downregulation in the expression of nuclear factor E2–related factor 2 (Nrf2) and glutathione peroxidase 4 (GPX4), and reduction of iron transport protein 1 (FPN1) expression by busulfan, which leads to oligospermia (Zhao et al. 2020). PM2.5 interferes with the metabolism of glutathione and purines, causing redox imbalance and the excessive accumulation of lipid peroxides; this induces the DNA damage response and cell cycle arrest in mice and leads to iron overload and ferroptosis, ultimately causing the impaired proliferation and decreased function of spermatogonia (Shi et al. 2022). Additionally, clinical studies have confirmed that the reduced expression of GPX4 and SLC7A11 and increased ferroptosis are associated with impaired sperm function in individuals with asthenozoospermia (Hao et al. 2023). In contrast, the relationship between impaired spermatogenic function and ferroptosis in juveniles due to PM2.5 has been little studied.

It is well established that oxidative stress can lead to cellular ferroptosis, and vitamins are the most common, readily available, and widely used antioxidants. Previous studies by our group demonstrated that combining low-dose vitamins did not have significant cytotoxic effects but had significantly enhanced antioxidant effects compared to their use alone (Liu et al. 2020).

Therefore, this study was carried out based on previous research. In this study, we focused on mitochondrial injury–mediated ferroptosis using immature testes as the research object and oxidative stress as the breakthrough point to investigate the effects of PM2.5 from automobile exhaust on the spermatogenic function of juvenile male rats, as well as the relationship between the reproductive toxic effects of PM2.5 and the impairment of mitochondrial function and morphology and ferroptosis in spermatogenic tissues. We also investigated whether the combination of vitamins could reduce the spermatogenic toxic effects of PM2.5 and explored the mechanisms of PM2.5-induced spermatogenic dysfunction in males.

## Materials and methods

### PM2.5 collection and preparation

The average concentration of PM2.5 should be less than 75 μg/m^3^ in 24 h as stipulated by the latest Ambient Air Quality Standards (GB3095-2012) in China. Based on the results obtained from our previous collection and purification of automobile exhaust particles using a Thermo Andersen atmospheric particulate sampler (GV2630, USA) and identification of the composition (Liu et al. 2019); the dose of PM2.5 in this study was set at 5 mg/kg, which corresponds to severe pollution as per China’s environmental standard (Liu et al. 2020; Liu et al. 2019).

In accordance with the experimental requirements, PM2.5 was configured to 10 mg/ml with sterile saline within 24 h prior to tracheal intubation and placed on a constant-temperature shaker at 37 °C and 60 rpm overnight.

### Vitamin intervention

Low-dose vitamins are known as antioxidants and are able to resist oxidation and protect cell function (Shen et al. 2018). Moreover, we previously confirmed that there was no significant cytotoxic effect of the combined use of low-dose vitamins, which has an obvious synergistic effect compared with single use (Liu et al. 2020). Therefore, low-dose vitamins (100 mg/kg vitamin C and 50 mg/kg vitamin E) were used in this experiment (Chakraborty and Jana 2017; Wassall et al. 2013).

### Animals and treatments

The experimental animals were purchased from the Animal Experiment Center of Zunyi Medical University (experimental facility certificate number/experimental animal use permit number: SYXK(Qian)2021-0004). All project procedures were approved by the ethical review board of experimental animal welfare of Zunyi Medical University. Sixty 21-day-old SPF-grade male SD rats weighing 70–100 g were selected and transferred to a barrier system room with an autonomous diet, 12-h light and dark cycles, 55 ± 5% humidity, 25 ± 2 °C room temperature, ventilation 8–12 times/hour, and acclimatization feeding for 1 week.

Twenty-eight-day-old male SD rats (postnatal day 28 (PND28)) were divided into a blank control group, vitamin control group, PM2.5 group (5 mg/kg), and PM2.5+Vitamin group (vitamin C 100 mg/kg, vitamin E 50 mg/kg). Fifteen rats were included per group. Rats in the blank control group were fed normally and without any special treatment. Based on normal feeding, intragastric administration of vitamins (100 mg/kg vitamin C and 50 mg/kg vitamin E, once a day), exposure to PM2.5 via tracheal cannula (once a day for 5 days a week, with a rest for 2 days), and exposure to PM2.5 via trachea cannula + intragastric administration of vitamins were respectively performed for the other groups for 4 weeks (Liu et al. 2020; Liu et al. 2019).

### Sample collection and sperm and fertility rate count

After the rats were subjected to the mating test during the period of sexual maturity (PND60); after their cervical disarticulation execution, the sperm filtrate was prepared by taking the tail of one side of the epididymis and placing it in saline at 37 °C, placing 10-μL drops of the suspension on a Towmax blood cell counting plate (25 × 16), and observing five fields of view for sperm counting with a light microscope. Furthermore, the fertility status of rats in each group at a later stage was recorded. The remaining tissue was frozen in liquid nitrogen and stored at −80 °C.

### Histological assessment of testes

The testicular tissues were washed with phosphate-buffered saline (PBS) and fixed with 10% paraformaldehyde for 48 h, following which samples were fixed, embedded, and then cut into 4-μm slices that were then deparaffinized, hydrated, stained with hematoxylin and eosin, and photographed with the image acquisition system of a light microscope (200× and 400×).

### Electron microscopy

After being cut into a size of about 1 mm^2^, the testicular tissues were fixed in 3% glutaraldehyde for more than 4 h. Next, they were rinsed with 0.1M PBS, re-fixed with 1% osmium tetroxide, washed, and subjected to successive dehydration with acetone. Thereafter, the dehydrated testicular samples were successively placed in solutions of a dehydrating agent and epoxy resin having ratios of 3:1, 1:2, and 1:3, with each step lasting for 30 to 60 min. After embedment and truncation, ultrathin sections with lengths of about 60 to 90 nm were prepared with an ultramicrotome, placed on copper mesh, and stained with uranium acetate followed by lead citrate at room temperature. Finally, the sections were observed and photographed using a transmission electron microscope.

### Western blotting

The protein extracts prepared after sample collection were detected by WB. The oxidative stress–associated protein nuclear factor–erythroid 2-related factor-2 (Nrf-2; ab92946, Abcam), lipid peroxidation–associated protein malondialdehyde (MDA; ab27642, Abcam), mitochondrial injury–associated protein cytochrome c oxidase IV (COXIV; ab202554, Abcam), heat shock protein 60 (HSP60; ab190828, Abcam), system Xc^−^–associated protein solute carrier family 7, member 11 (SLC7A11; ab175186, Abcam), glutathione peroxidase-4 (GPX4; ET1706-45, HUABIO), ferroptosis-associated protein ferroportin-1 (FPN1; ab239511, Abcam), and divalent metal transporter 1 (DMT1; ab157208, Abcam) were used, with β-actin (AF7018, Affinity, England) as the control. Goat anti-rabbit IgG (Biosharp, China) was used as the secondary antibody. A western blotting ECL kit (ZETA-Life, San Francisco, CA, USA) was used. After the detection step, protein bands were observed and photographed using an automated chemiluminescence image analysis system (Tanon, Shanghai, China), and various parameters were quantified using ImageJ software (National Institutes of Health, DC, USA). The procedure was repeated three times before statistical analysis using SPSS 26.0.

### Measurement of the levels of total iron ions and ferrous ions

After sample collection, the testicular tissues (10 mg) were placed in buffer on ice for homogenization and centrifuged (12,000×g, 10 min) to obtain a clear supernatant for assay. The supernatant was incubated with an iron probe and measured using a microplate reader at a wavelength of 593 nm. The levels of ferrous ions and total iron ions in the testes were respectively measured using colorimetric assay kits for ferrous ions (E-BC-K773-M, Elabscience) and total iron ions (E-BC-K772-M, Elabscience).

### Statistical analysis

SPSS26.0 software was employed for statistical analysis. The normality test and *F*-test for homogeneity of variance were performed, and inter-group comparison was made using one-way analysis of variance. All data were expressed as mean standard ± deviation, and *p* < 0.05 suggested a statistically significant difference. GraphPad Prism 9.0 was used to generate statistical graphs.

## Results

### Changes of sperm, fertility rate, and testis histology in juvenile male rats during the period of sexual maturity after PM2.5 exposure

Observation of the testicular tissues of the male rats in the PM2.5 group under a light microscope revealed a decrease in the number of sperms in the testes. Moreover, the fertility rate of the rats in the PM2.5 group significantly declined after they were subjected to the mating test. In the PM2.5+Vitamin group, there was an increase in the number of sperms in the testes compared with the PM2.5 group, and the fertility rate rose after the rats were subjected to the mating test (Tables 1 and 2, *p* < 0.05).

**Table 1:** Analysis of sperm amount in each group after PM 2.5 exposure

Group	Number	Sperm count (×10^6^/ml)
Control	15	54.50 ± 2.396
Vitamin	15	57.86 ± 1.055
PM2.5	15	34.32 ± 2.978*
PM2.5+Vitamin	15	44.40 ± 1.059

Numbers are expressed as the mean standard ± deviation (*n* = 15)

*p < 0.05 *vs.* control group

**Table 2:** Mating test in each group after PM 2.5 exposure

Group	Male	Female	Conception	Conception rate (%)
Control	15	15	14	93.33%
Vitamin	15	15	15	100%
PM2.5	15	15	9	66%
PM2.5+Vitamin	15	15	12	80%

Numbers are expressed as the mean standard ± deviation (*n* = 15)

*p < 0.05 *vs.* control group

**Fig. 1:**
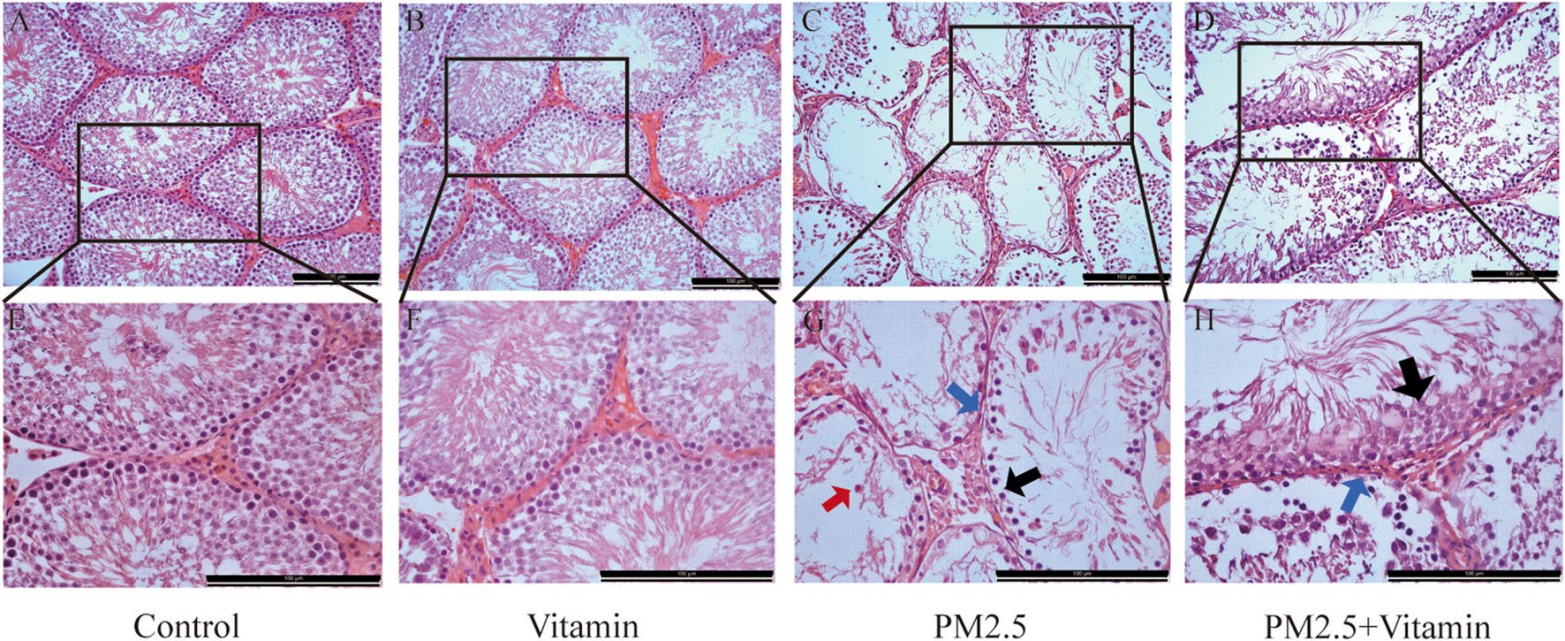
Histological change in the testes after PM 2.5 exposure. In the blank control group and vitamin control group (**A**, **E**, **B**, **F**), the spermatogenic cells in the seminiferous tubules are in alignment, with a large number of sperms accumulating in the lumen. In the PM2.5 group (**C**, **G**), the epithelium of the seminiferous tubules is significantly atrophic (the blue arrow), the number of spermatogenic cell layers and sperms in the seminiferous tubules distinctly decreases (the black arrow), and some spermatogenic cells fall to the lumen (the red arrow). In the vitamin intervention group (**D**, **H**), the morphology of seminiferous tubules gradually recovers (the blue arrow), with a significant increase in the number of spermatogenic cell layers (the black arrow) and an increase in spermatogenesis. The upper row is 200×, the lower row is 400×, and the proportional scale is 100 μm

Observation of the testicular tissues of male rats in the PM2.5 group under an electron microscope showed significant atrophy of the epithelium of the seminiferous tubules, a distinct decrease in the number of spermatogenic cell layers and sperms in the seminiferous tubules, detachment of some spermatogenic cells into the lumen, and an increase in the number of abnormal sperms. In the PM2.5+Vitamin group, the morphology of the seminiferous tubules gradually recovered, the number of spermatogenic cell layers rose significantly, and spermatogenesis increased (Fig. 1).

### Oxidative-antioxidant imbalance in the testes of juvenile male rats during the period of sexual maturity after PM2.5 exposure

**Fig. 2:**
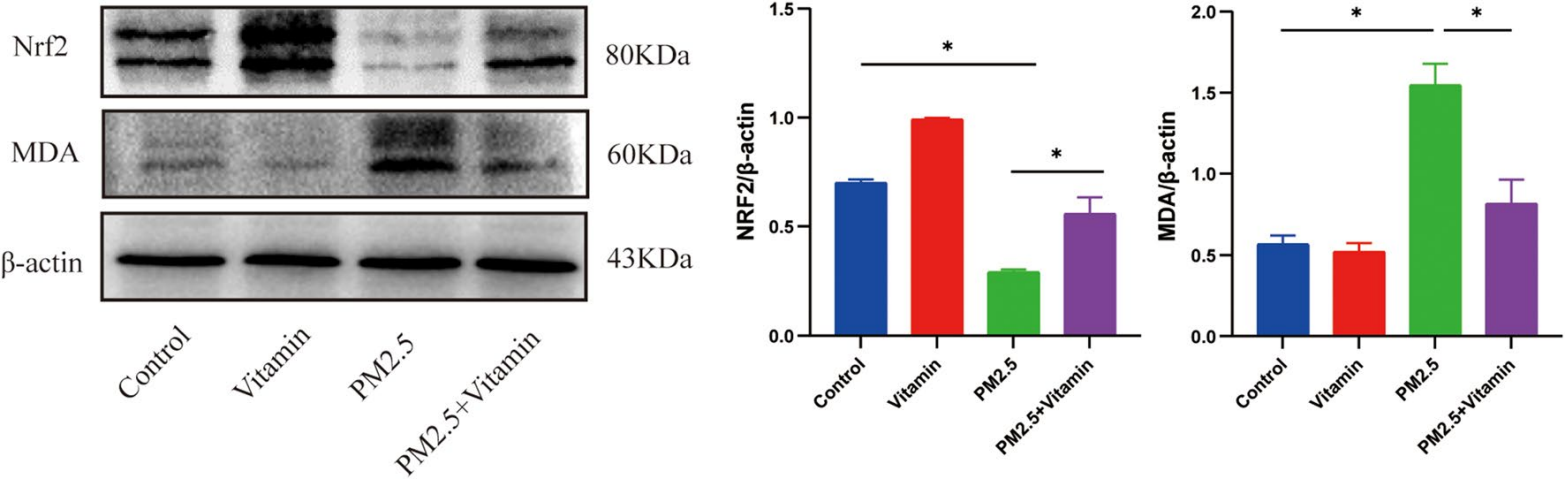
ROS level in the testes after PM 2.5 exposure. The expression level of Nrf2 is significantly lower in the PM2.5 group than in the control group; meanwhile, the expression level of Nrf2 increased sharply in the vitamin intervention group than in the PM2.5 group. On the contrary, the expression level of MDA is significantly higher in the PM2.5 group than in the control group, the expression level of MDA decreased sharply in the vitamin intervention group than in the PM2.5 group. *p < 0.05, **p < 0.01

WB results showed a significant decrease in the expression level of the oxidative stress-associated protein Nrf2 in the PM2.5 group, which increased distinctly in the PM2.5+Vitamin group. There was a marked increase in the expression level of the lipid peroxidation-associated protein MDA in the PM2.5 group, which decreased in the PM2.5+Vitamin group (Fig. 2, *p* < 0.05).

### Mitochondrial damage in the testicular cells of juvenile male rats during the period of sexual maturity after PM2.5 exposure

**Fig. 3:**
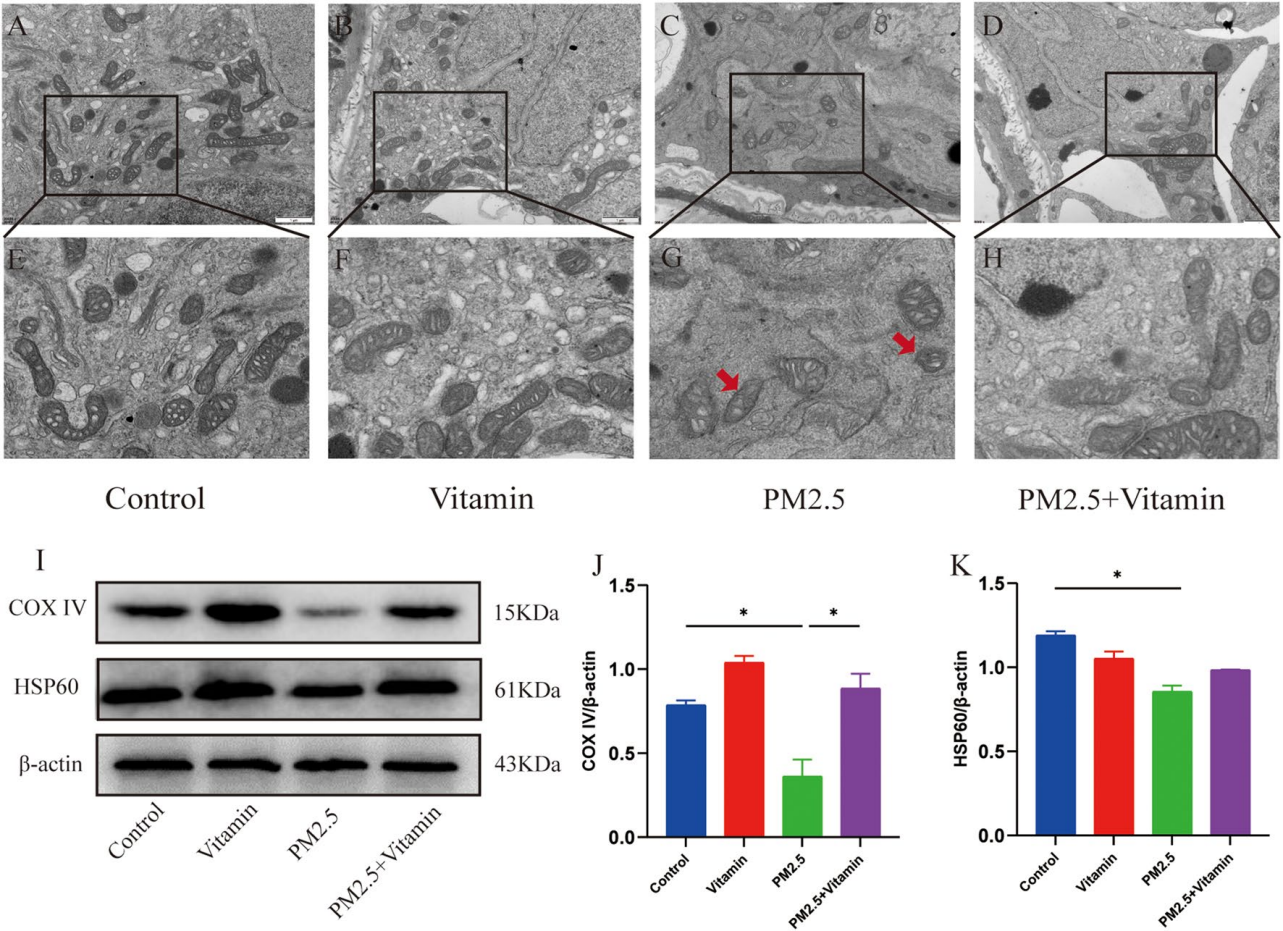
Mitochondrial damage in testicular cells after PM 2.5 exposure. In the blank control group (**A**, **E**) and vitamin control group (**B**, **F**), the cell morphology is normal, with irregular nuclei, and chromatin (mainly euchromatin) is evenly distributed. Mitochondria, rough endoplasmic reticulum, and other organelles with complete and clear structures as well as a small number of primary lysosomes are observed in the cytoplasm. In the PM2.5 group (**C**, **G**), some mitochondria show reduced volume, atrophied membrane, and decreased cristae (the red arrow). In the vitamin intervention group (**D**, **H**), the mitochondrial volume and cristae increase and mitochondrial membrane atrophy is alleviated compared with the PM2.5 group. **I**–**K** The changes in the expression levels of proteins related to mitochondrial damage. The levels of HSP60 and COXIV are significantly lower in the PM2.5 group than in the control groups but higher in the vitamin intervention group than in the PM2.5 group. *p < 0.05, **p < 0.01

Observation of mitochondria in the testicular cells under an electron microscope showed mitochondrial volume reduction, membrane atrophy, and cristae decrease in the PM2.5 group and alleviated mitochondrial damage in the PM2.5+Vitamin group. Meanwhile, WB results showed a significant decrease in the expression levels of mitochondrial injury-related proteins COXIV, and HSP60 was also somewhat elevated but there was no statistical significance, which rose distinctly in the PM2.5+Vitamin group (Fig. 3, *p* < 0.05).

### Metabolic disorder of iron ions in the testicular tissues of juvenile male rats during the period of sexual maturity after PM2.5 exposure

**Fig. 4:**
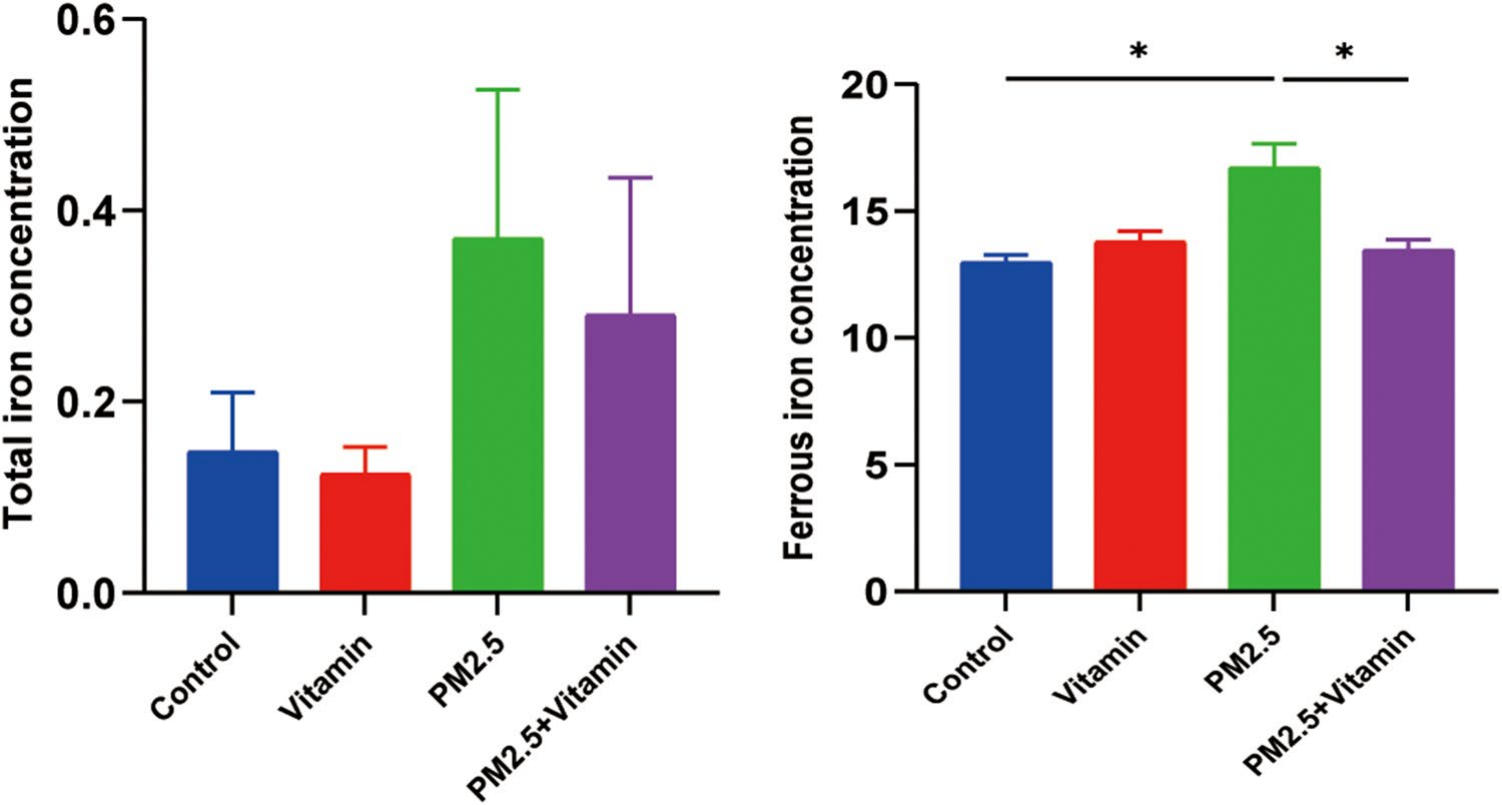
Metabolic disorder of iron ions in testicular tissues after PM 2.5 exposure. The concentrations of Fe^2+^ and Fe^3+^ are significantly higher in the PM2.5 group than in the control groups but lower in the vitamin intervention group than in the PM2.5 group. *p < 0.05, **p < 0.01

The results of the detection of total iron ions and ferrous ions using colorimetric assay kits showed that the numbers of total iron ions and ferrous ions in the testicular tissues of rats in the PM2.5 group were higher than the corresponding numbers in the normal group; the increase in total iron was not statistically significant, but the increase in ferrous ions was statistically significant. In the PM2.5+Vitamin group, the numbers of total iron ions and ferrous ions both decreased to a certain extent (Fig. 4, *p* < 0.05).

### Ferroptosis in the testicular tissues of juvenile male rats during the period of sexual maturity after PM2.5 exposure

**Fig. 5:**
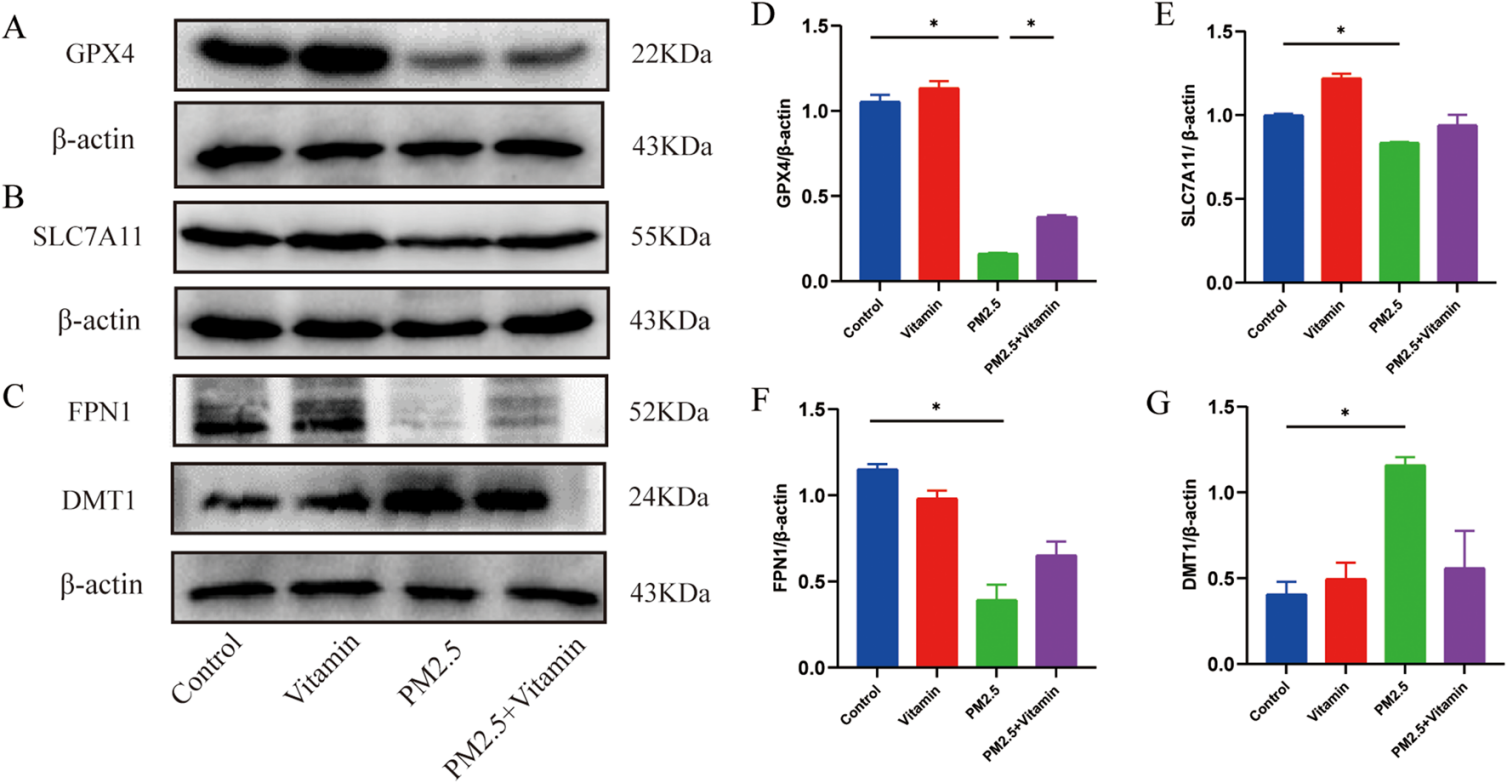
Ferroptosis in testicular tissues after PM 2.5 exposure. The expression of GPX4 is significantly lower in the PM2.5 group than in the control groups but higher in the vitamin intervention group than in the PM2.5 group (**A**, **D**). The expression of SLC7A11 significantly decreases in the PM2.5 group compared with the control groups but is higher in the vitamin intervention group than in the PM2.5 group (**B**, **E**). The expression of FPN1 in the PM2.5 group is significantly lower than in the control group, but the level in the vitamin intervention group is higher than that in the PM2.5 group (**C**, **F**). The expression of DMT1 in the PM2.5 group is significantly higher than in the control group, but the level in the vitamin intervention group is lower than that in the PM2.5 group (**C**, **G**). The data are described as mean ± standard deviation, and all experiments were repeated at least 3 times. *p < 0.05, **p < 0.01

In the PM2.5 group, the expression level of the phospholipid hydroperoxide glutathione peroxidase GPX4 significantly decreased but rose distinctly in the PM2.5+Vitamin group, and the expression level of cystine-glutamate transporter system Xc^−^–associated protein SLC7A11 declined but rose to a certain extent in the PM2.5+Vitamin group. The expression level of FPN1 distinctly decreased but significantly increased in the PM2.5+Vitamin group, and the expression level of DMT1 rose significantly but declined distinctly in the PM2.5+Vitamin group (Fig. 5, *p* < 0.05).

## Discussion

In this study, after juvenile male rats were exposed to PM2.5 for 4 weeks, their reproductive functions during the period of sexual maturity were studied using six parameters, namely, the sperm count, fertility rate, histological morphology, mitochondrial morphology, iron ion concentration, and ROS, mitochondrial damage, and ferroptosis relational protein expression levels. The results showed that compared with rats in the control group, juvenile male rats exposed to PM2.5 had a decrease in the number of sperms and the fertility rate during the period of sexual maturity. Additionally, obvious atrophy of the epithelium of the seminiferous tubules, a distinct decrease in the number of spermatogenic cell layers and sperms in the seminiferous tubules, the detachment of some spermatogenic cells into the lumen in the testes of male rats during the period of sexual maturity were observed. These findings confirm that the reproductive function of juvenile male rats during the period of sexual maturity decreased after PM2.5 exposure, which enriches our previous research (Liu et al. 2020; Liu et al. 2019).

Our previous studies confirmed that oxidative stress is one of the important mechanisms by which PM2.5 induces reproductive toxicity in adult male rats (Liu et al. 2020; Liu et al. 2019). The decrease in the number of sperms in adult male rats may be partly attributed to systemic and testicular localized inflammatory responses caused by PM2.5 exposure during adolescence (Zhou et al. 2019). Such a decrease may also be ascribed to the decreased testosterone concentration and hindered testosterone biosynthesis, which further affect sperm concentration, morphology, and viability (Yang et al. 2019). Some scholars have found that PM2.5 may lead to reproductive impairment by inducing decreased sperm quality and testosterone levels in juvenile male rats via the inositol-requiring enzyme (IRE1)/c-Jun NH (2)-terminal kinase (JNK)/autophagy signaling pathway (Yang et al. 2021). Additionally, after mother rats are exposed to PM2.5, PM2.5 may affect spermatogenesis by activating the unfolded protein response (UPR)–mediated JNK apoptosis pathway in the testes of their offspring to increase apoptosis and reduce testosterone secretion (Ren et al. 2022). Based on previous studies, we found that the expression level of antioxidant transcription factor Nrf2 significantly decreased, while that of lipid peroxide MDA distinctly increased in the testes of juvenile male rats during the period of sexual maturity after PM2.5 exposure, which again verified that oxidative stress is the initiating agent for PM2.5 to induce reproductive damage in male rats and that the redox is imbalanced and lipid peroxides are accumulated in the testes of juvenile male rats during the period of sexual maturity after PM2.5 exposure.

As the centers of energy metabolism in cells, mitochondria play an important role in maintaining cell homeostasis (Yang et al. 2016). However, mitochondria may be damaged due to increased ROS (Zhao et al. 2021), leading to abnormal cell death. Our previous work confirmed that PM2.5 can cause mitochondrial dysfunction by damaging the UPR^mt^ pathway of mitochondria in spermatogenic cells, which further leads to spermatogenic impairment in juvenile rats (Wang et al. 2022). Therefore, we further investigated mitochondrial damage. COXIV is considered a key marker of mitochondrial function because of its rate-limiting effect on oxidation (Holper et al. 2019; Srinivasan and Avadhani 2012), while HSP60 plays a key role in regulating mitochondrial protein homeostasis (Duan et al. 2020). Therefore, through the detection of the expression levels of associated proteins in the testicular tissues of juvenile male rats during the period of sexual maturity after PM2.5 exposure, we discovered a significant decrease in the expression levels of COXIV and HSP60 and observed reduced volume, atrophied membrane, and cristae decrease in some mitochondria of testicular cells via electron microscopy, which indicated that mitochondria in the testicular tissues of juvenile male rats during the period of sexual maturity are seriously damaged after PM2.5 exposure. Studies have confirmed that mitochondria regulate cysteine metabolism through the tricarboxylic acid (TCA) cycle and the electron transport chain (ETC) and that disruption of TCA metabolism, ETC activity, and mitochondrial membrane potential (MMP) hyperpolarization resulting in cysteine deficiency cause lipid ROS accumulation, which in turn leads to cellular ferroptosis following mitochondrial damage (Gao et al. 2019).

Ferroptosis is a novel mode of cell death depending on iron ions, triggered by the excessive accumulation of the toxicity of lipid peroxides on the cell membrane (Dixon et al. 2012; Lei et al. 2022; Stockwell et al. 2017; Wu et al. 2019). Some scholars have confirmed that PM2.5 leads to pulmonary fibrosis by inducing ferroptosis through the transforming growth factor beta (TGF-β) signaling pathway (Guo et al. 2022) and causes decreased reproductive function in adolescent male rats by inducing DNA damage and ferroptosis through oxidative stress (Shi et al. 2022). When ferroptosis occurs in cells, mitochondrial damage is manifested as volume reduction, membrane atrophy, and cristae decrease or disappearance (Dixon et al. 2012; Xie et al. 2016). We observed these manifestations in the mitochondria of testicular cells in juvenile male rats during the period of sexual maturity after PM2.5 exposure, suggesting that ferroptosis also occurs in this experimental model.

To confirm the presence of ferroptosis, we further examined the iron ion content in testicular tissue; the results showed that male rats in the PM2.5 group had higher concentrations of total iron ions and ferrous ions and a metabolic disorder in the testicular tissues during the period of sexual maturity compared with rats in the normal group, further confirming that ferroptosis occurs in this experimental model.

Furthermore, ferroptosis is caused by the accumulation of cellular ROS exceeding the contents of glutathione (GSH) and phospholipid hydroperoxide peroxidase with GSH as the substrate in redox reactions and can be triggered by inhibiting the activity of System Xc^−^, leading to the depletion of cellular cysteine and GSH and redox homeostasis imbalance in cells (Gao et al. 2019). Some researchers found that the expression level of GPX4 significantly declined in infertile men diagnosed with azoospermia (Imai et al. 2009; Yang et al. 2014). On this basis, we evaluated the expression levels of phospholipid hydroperoxide GPX4 and system Xc^−^–associated protein SLC7A11 in the testicular tissues during the period of sexual maturity. The results showed that the expression levels of GPX4 and SLC7A11 in the testicular tissues decreased, indicating reduced ROS capacity, decreased activity of system Xc^−^, and redox imbalance in the testicular tissues of juvenile male rats during the period of sexual maturity after PM2.5 exposure, which were consistent with the manifestations of ferroptosis.

FPN1, the only transmembrane protein found to be related to the release of iron ions in mammals so far, is responsible for exporting ferrous ions from cells to the plasma (Tian et al. 2021). The ferrous ion transfer protein DMT1 can regulate iron ion levels and is crucial for maintaining iron homeostasis (Song et al. 2021). Therefore, FPN1 and DMT1 are vital for iron ion homeostasis. After confirming the metabolic disorder of iron ions in this model, we further studied changes in the expression levels of proteins related to ferroptosis using WB. Compared with the normal group, the PM2.5 group displayed a significantly decreased level of FPN1 and a distinctly increased DMT1 level in the testicular tissues, proving that ferroptosis in the testicular tissues of juvenile male rats during the period of sexual maturity after PM2.5 exposure rises significantly, leading to impaired spermatogenic function.

Oxidative stress is known to be an “initiating agent” for PM2.5 to induce reproductive impairment in adult male rats, and the combined use of vitamins E and C can reduce oxidative stress by inhibiting the p38 MAPK signaling pathway and blocking DEHP-induced spermatogenic dysfunction and the destruction of the blood-testis barrier (Shen et al. 2018). Thus, we added the vitamin intervention group to the model to investigate whether the early enhancement of the antioxidant capacity of juvenile male rats can reduce the reproductive toxicity of PM2.5. The results revealed an increase in spermatogenesis and a reduction in oxidative stress, mitochondrial damage, and ferroptosis in the testes of male rats during the period of sexual maturity in the PM2.5+Vitamin group compared with the PM2.5 group. In addition, the fertility rate of the rats rose significantly after they were subjected to the mating test.

Therefore, this study confirmed that the reproductive toxicity of PM2.5 in juvenile male rats was closely related to oxidative stress, mitochondrial damage, and ferroptosis. However, further research is needed to determine the specific underlying mechanism and target of action of oxidative stress leading to ferroptosis. Moreover, further studies are required regarding whether reproductive function can recover spontaneously and the recovery degree after rats are removed from the environment with PM2.5.

## Conclusion

This study showed for the first time that the exposure of juvenile male rats to PM2.5 can lead to a severe decline in their reproductive function during the period of sexual maturity, the reproductive toxicity of PM2.5 is closely related to oxidative stress and ferroptosis, and the early administration of vitamins can enhance the antioxidant capacity of juvenile male rats, thus reducing the reproductive toxicity of PM2.5 in juvenile male rats.
